# Deafness and early language deprivation influence arithmetic performances

**DOI:** 10.3389/fnhum.2022.1000598

**Published:** 2022-11-30

**Authors:** Margot Buyle, Virginie Crollen

**Affiliations:** Psychological Sciences Research Institute (IPSY) and Institute of Neuroscience (IoNS), Université catholique de Louvain, Louvain-la-Neuve, Belgium

**Keywords:** deafness, arithmetic, subtraction, multiplication, sign language, verbal, visuospatial

## Abstract

It has been consistently reported that deaf individuals experience mathematical difficulties compared to their hearing peers. However, the idea that deafness and early language deprivation might differently affect verbal (i.e., multiplication) vs. visuospatial (i.e., subtraction) arithmetic performances is still under debate. In the present paper, three groups of 21 adults (i.e., deaf signers, hearing signers, and hearing controls) were therefore asked to perform, as fast and as accurately as possible, subtraction and multiplication operations. No significant group effect was found for accuracy performances. However, reaction time results demonstrated that the deaf group performed both arithmetic operations slower than the hearing groups. This group difference was even more pronounced for multiplication problems than for subtraction problems. Weaker language-based phonological representations for retrieving multiplication facts, and sensitivity to interference are two hypotheses discussed to explain the observed dissociation.

## Introduction

A converging body of evidence in the numerical cognition field suggests that different arithmetic operations rely on distinct neuro-cognitive processes. Indeed, while subtraction is solved using visuospatial procedures (Dehaene, 1992; Campbell and Xue, 2001; Robinson, 2001; Seyler et al., 2003; Thevenot and Barrouillet, 2006; Barrouillet et al., 2008; Prado et al., 2014) and visuospatial shifts of attention (Li et al., 2018; Salvaggio et al., 2022) multiplication is, in contrast, rote learnt and stored in verbal memory (Verguts and Fias, 2005). Visuospatial skills accordingly predict subtraction, but not multiplication operations. Language skills inversely predict multiplication but not subtraction (Lee and Kang, 2002; Guez et al., 2022). At the neural level, subtraction has been linked to an increased activity of the parietal cortex, typically associated with quantity and visuospatial processing. Multiplication, on the other hand, relies on verbal brain areas of the left hemisphere (Lee, 2000; Zhou et al., 2007; Prado et al., 2011). In neuropsychology, impairments in phonological processing (e.g., dyslexic individuals) induce marked difficulties in multiplication fact retrieval but no impairment in subtraction (Simmons and Singleton, 2008; Boets and De Smedt, 2010; De Smedt and Boets, 2010). Double dissociations have moreover been reported with some patients selectively impaired in subtraction (Dehaene and Cohen, 1997; van Harskamp and Cipolotti, 2001) and others presenting the exact opposite pattern of performance: a selective impairment in multiplication fact retrieval and a preservation of their subtraction performances (Cohen and Dehaene, 2000; Cappelletti et al., 2001; van Harskamp and Cipolotti, 2001; Sandrini et al., 2003).

When taking the link that exists between language skills and arithmetic processing into account, it is not surprising to see that deaf individuals, who often experience some level of language deprivation in early childhood, present poorer numerical abilities than their hearing peers (see Buyle et al., 2021 for a review). A delay of 2 to 3.5 years on mathematical achievement tests (Nunes and Moreno, 2002; Bull et al., 2005) has indeed been highlighted and appears to be more pronounced in verbal numerical tasks (e.g., see Nunes et al., 2009 for multiplicative reasoning; Andin et al., 2014 for relational statements, Serrano Pau, 1995; Titus, 1995; Kelly et al., 2003 for fractions) than in visuospatial numerical tasks. In line with this, the absence of the SNARC effect in a verbal parity judgement task vs. the presence of the SNARC effect in a visuospatial number comparison task was recently shown in one of our previous studies (Buyle et al., 2022).

These observations were interestingly assumed to be caused by some linguistic aspects (Serrano Pau, 1995; Kelly and Mousley, 2001; Kelly et al., 2003; Pagliaro, 2010). In contrast to oral languages, sign languages are formed by several visual components such as the configuration, movement, orientation and location of the hands in space, the body posture, the facial expression and the movement of the mouth (Emmorey, 2002; Sandler and Lillo-Martin, 2006). These visual and motor aspects of sign language have already been shown to impact cognitive processes such as memory (Wilson and Emmorey, 1997) and reading (Quandt and Kubicek, 2018). Alpha and Beta EEG signals were for example found to be different when deaf signers read English words whose American Sign Language translations use two hands vs. one hand (Quandt and Kubicek, 2018). This result demonstrates the involvement of the sensorimotor system in cross-linguistic translation and supports the Dual-Route Cascade (DRC) model proposed by Elliott et al. (2011). This model suggests that the cognitive system involved in reading is fundamentally the same in deaf as in hearing (see the DRC model of Coltheart et al., 2001), but the types of activated units are different: visemes and phonemes for multimodal deaf bilingual vs. phonemes for monolingual or unimodal bilingual hearing individuals. L1 and L2 lexicons are both activated when deaf signers are reading. The viseme-phoneme translation that occurs in deaf signers can therefore affect their reading speed and proficiency. Associations between sign phonology and reading skills (Mayberry et al., 2011; Rudner et al., 2012) were accordingly reported in deaf individuals (Davis and Kelly, 2003).

As a close correlation between phonological awareness and arithmetic problem solving has also been repeatedly observed (De Smedt et al., 2010), the parallel between reading and arithmetic is tempting. The fact that sign languages use the entire body in a spatial-visual-somatic way may, for example, preserve or even positively impact (Chinello et al., 2012) the visuospatial arithmetic abilities of deaf individuals. In contrast, the fact that deaf signers do not easily access the phonology of verbal languages or access it through a viseme-phoneme translation may, in contrast, negatively impact their verbal arithmetic abilities (as already observed in reading, see Elliott et al., 2011). While this hypothesis is tempting, recent studies nevertheless failed to demonstrate clear results supporting this claim. While Andin et al. (2014) demonstrated that deaf signers perform worse on multiplication than on subtraction operations, more recent studies failed to demonstrate this dissociation (Andin et al., 2019). Mixed conclusions can also be found at the brain level. While an fMRI study showed that the right horizontal intraparietal sulcus was more activated in deaf signers as compared to hearing during multiplication operations (Andin et al., 2019), more recent studies (Andin et al., 2022; Berteletti et al., 2022) highlighted a comparable dissociation between the brain networks supporting multiplication and subtraction in deaf and hearing participants. There is therefore an urgent need to better characterise the impact deafness and its related language experience may have on arithmetic processing.

To do so, we will ask Belgian deaf signers, hearing signers and hearing controls to perform easy and difficult subtraction and multiplication operations. In Belgium, there are few options regarding education of deaf children. First, there exists the special-need education schools, but sign language is not provided as instruction language since the teachers are often hearing and using spoken language. Second, there is the regular school system with the presence of a sign language interpreter. However, the deaf child has to be confident with sign language before he/she can benefit from “translated” classes. A third and last option is the bilingual-bicultural education, which offers deaf children all the opportunities to get into contact with both spoken and signed languages, and both cultures. Unfortunately, not many schools provide this educational system in Belgium. Many Belgian deaf signers therefore consider sign language as their preferred communication method but were taught arithmetic in another spoken language. Including hearing signers in this study will therefore allow us to examine whether the arithmetic difficulties experienced by deaf signers are merely linked to the use of sign language or to the use of sign language as mother tongue (L1) while being taught arithmetic in a second spoken language (L2: Dutch or French). As several studies already demonstrated that unimodal bilingualism can impact number and arithmetic processing (Van Rinsveld et al., 2015, 2016, 2017; Lachelin et al., 2022), there is no reason to believe that number transcoding in bimodal bilinguals could not have any impact on arithmetic performances.

Finally, as recent behavioural (De Visscher and Noël, 2014a,b) and brain (De Visscher et al., 2018) findings on hearing people suggest that individual differences in multiplication fact knowledge may be partly due to differences in sensitivity to interference (De Visscher and Noël, 2013), we also decided to investigate this concept. It is based on the interference model of Campbell (1987) and Campbell (1995) according to which arithmetic facts involve various combinations of the digits 0 to 9, and therefore consist of very similar associations between two operands and the answer. As the similarity between the items to remember can cause memory interference (Oberauer and Lange, 2008), learning arithmetic facts that share a lot of common features can therefore be considered as highly interfering for the memory (Wickelgren, 1979). Individuals with higher sensitivity to interference therefore experience more proactive overlap from previously learned problems during arithmetic fact retrieval (De Visscher et al., 2018). A central executive impairment can therefore cause difficulties in arithmetic fact retrieval (Kaufmann, 2002; Temple and Sherwood, 2002; Noël et al., 2004; Barrouillet and Lépine, 2005), especially when a deficit in suppressing irrelevant information is present (i.e., inhibition) (Barrouillet et al., 1997; Pasolunghi et al., 1999; Censabella and Noël, 2004; Passolunghi and Siegel, 2004; Geary et al., 2012). De Visscher and Noël (2013) for example reported a case study of a dyscalculic individual showing hypersensitivity to interference in memory, and a circumscribed impairment to store arithmetic facts. Although deaf children and adults were often reported to present lower executive functioning than their hearing peers (Figueras et al., 2008; Hauser et al., 2008; Hintermair, 2013; Dye and Hauser, 2014; Hall et al., 2016; Botting et al., 2017; Jones et al., 2020), their sensitivity to interference while performing single-digit multiplication problems was never taken into account. This will be done in the present study.

To sum up, our study aims to investigate whether the arithmetic deficit in deaf individuals is: (1) global, or more specifically related to verbal numerical operations (i.e., multiplication problems); (2) linked to auditory deprivation, language deprivation, the mere use of sign language or the use of sign language as L1 while being taught arithmetic in a spoken L2 (Dutch or French that might not have been fully accessible despite the use of hearing aids); and (3) linked to the interference index of single-digit multiplication problems. If the arithmetic difficulties of deaf adults are global, their performance should be worse than the one of the hearing signers and hearing controls in both arithmetic operations. If deafness and its related language experience more strongly affects verbal operations, the difference between the deaf and the hearing adults should be bigger for the multiplication operations. Finally, as hearing signers were taught arithmetic in their mother tongue (i.e., French or Dutch), their later acquisition of sign language should not affect their arithmetic performances. They should therefore behave exactly as the hearing controls.

## Methods

### Participants

Three groups of 21 adults were recruited in the Dutch and French-speaking parts of Belgium: a group of congenitally deaf adults (12 females, 10 French, Mage = 39.1 years ± 2.92), a group of hearing signers (16 females, 11 French, Mage = 37.6 years ± 2.95), and a control group of hearing adults who did not know sign language (12 females, 10 French, Mage = 38.8 years ± 3.15) (see Table 1 for a detailed description of the participants). All participants had normal or corrected-to-normal vision and no neurological problems. Hearing participants were matched to deaf participants for gender [*X*^2^ (2, 63) = 2.19, *p* = 0.33], age [*F*_(2, 60)_ = 0.066; *p* = 0.94, η^2^ = 0.002], educational level [*F*_(2, 58)_ = 2.230; *p* = 0.12, η^2^ = 0.071], handedness [*X*^2^ (2, 63) = 1.11, *p* = 0.58], and mother tongue (French vs. Dutch) [*X*^2^ (2, 63) = 0.13, *p* = 0.94]. Hearing signers reported a minimum level of B1 (i.e., intermediate CEFR level) for sign language (see Supplementary Table 1 for more details). Most (13) deaf individuals reported sign language as their mother tongue. Only seven deaf participants indicated being born in a deaf family, but six deaf participants indicated sign language as their mother tongue although not having any relatives with hearing problems. On the other hand, one deaf indicated having Dutch with gestures as mother tongue, and eight deaf participants reported acquiring sign language later in their life (2 to 20 years old), however, they were fluent in sign language and indicated it as their preferred way of communication (see Supplementary Table 2 for more details). Both oral and written instructions in Dutch and in French were given, as well as instruction videos in sign language for deaf participants. Questions could be asked to the researcher, who is basic proficient in sign language. When really experiencing a language barrier, questions were answered in a written manner. Participants provided their written informed consent and the procedures were in line with the Declaration of Helsinki. The study was approved by the “Comité d’Ethique Hospitalo-Facultaire Saint-Luc-UCLouvain” (2019/19AOU/357).

**TABLE 1:** Characteristics of participants.

Subject	Age	Sex	Handedness	Onset	Cause	Formal school years (after primary school)
1	56	F	R	0	Hereditary	13
2	47	M	L	0	Rubella	6
3	26	F	R	3 years	Meningitis	12
4	26	F	R	0–1 year	Unknown	7
5	48	M	R	0	O_2_ insufficiency	6
6	51	F	R	0	Meningitis	15
7	28	M	R	0	Genetic	14
8	50	M	L	0	Genetic	7
9	37	F	R	0	Genetic	12
10	49	F	R	0	Rubella	7
11	23	F	R	0	Unknown	9
12	43	M	R	0	Hereditary	5
13	24	F	R	0	Unknown	11
14	20	M	R	0	Unknown	7
15	53	M	R	0	Hereditary	6
16	53	F	R	0	Hereditary	6
17	35	M	R	0	Unknown	9
18	63	F	R	0	Hereditary	N/A
19	35	M	L	0	Genetic	8
20	37	F	R	0	Nerf atrophy	12
21	22	F	R	0	Unknown	10
22	21	F	R	0	CMV	8
23	23	F	R	/	/	9
24	30	F	R	/	/	12
25	23	F	R	/	/	11
26	31	F	R	/	/	N/A
27	58	F	R	/	/	12
28	23	F	R	/	/	7
29	26	F	R	/	/	12
30	29	F	R	/	/	9
31	51	F	R	/	/	17
32	32	F	R	/	/	11
33	41	F	R	/	/	9
34	23	F	R	/	/	8
35	29	F	L	/	/	15
36	57	F	L	/	/	14
37	28	F	R	/	/	16
38	30	F	R	/	/	11
39	50	M	R	/	/	10
40	63	M	R	/	/	9
41	51	M	R	/	/	10
42	54	M	R	/	/	6
43	38	M	R	/	/	12
44	55	F	R	/	/	11
45	23	M	R	/	/	12
46	23	M	R	/	/	9
47	23	F	R	/	/	10
48	20	F	R	/	/	8
49	38	F	R	/	/	12
50	50	M	R	/	/	11
51	57	F	R	/	/	6
52	46	M	R	/	/	6
53	66	M	R	/	/	11
54	31	M	R	/	/	16
55	57	M	R	/	/	10
56	50	F	R	/	/	9
57	39	M	R	/	/	8
58	38	F	R	/	/	9
59	25	F	R	/	/	14
60	36	F	L	/	/	11
61	49	M	R	/	/	7
62	47	F	R	/	/	7
63	20	F	R	/	/	9
64	21	F	R	/	/	10

R, right-handed; L, left-handed; F, female; M, male; CMV, cytomegalovirus.

### Task and procedure

Participants had to solve two different arithmetic problems, namely subtraction problems and multiplication problems, which were presented on a computer screen in black font (Courier New font and size 42) on a grey background. Each category of operations consisted of 20 problems to solve: some easy operations (i.e., without carry-over for subtraction; one-digit number × one-digit number for multiplication) and some difficult operations (i.e., with carry-over for subtraction problems; two-digit number × one-digit number for multiplication problems; see Table 2). The participants first had to press the space bar when they knew the answer (to collect correct reaction times), and then use the keyboard to write down their answers. Operations were presented in a fixed order starting with subtraction problems and then multiplication problems. Easy operations were also presented before the difficult ones. This was done to not discourage deaf participants who are known to experience difficulties with arithmetic (Hyde et al., 2003; Kelly et al., 2003; Bull et al., 2011). Operations were presented on the screen until the participant pressed the space bar to be able to indicate their answer. The accuracy and reaction times of the responses were measured. Subjects executed the task in a silent room where the task was presented and the responses were recorded using E-Prime 2.0 software running on a Dell computer with Windows XP as operating system.

**TABLE 2:** Operations presented to the participants.

Subtraction	Carryover	Level	Multiplication	Format	Level	Interference index
6–2	No	Easy	3 × 2	U × U	Easy	0
8–5	No	Easy	4 × 3	U × U	Easy	10
7–3	No	Easy	5 × 4	U × U	Easy	8
9–4	No	Easy	6 × 5	U × U	Easy	6
17–5	No	Easy	8 × 6	U × U	Easy	11
27–4	No	Easy	2 × 7	U × U	Easy	4
48–6	No	Easy	9 × 4	U × U	Easy	9
54–3	No	Easy	4 × 8	U × U	Easy	25
63–9	Yes	Difficult	7 × 9	U × U	Easy	17
35–6	Yes	Difficult	5 × 7	U × U	Easy	7
21–7	Yes	Difficult	13 × 5	DU × U	Difficult	N/A
44–8	Yes	Difficult	24 × 4	DU × U	Difficult	N/A
24–11	No	Easy	38 × 3	DU × U	Difficult	N/A
58–33	No	Easy	17 × 6	DU × U	Difficult	N/A
27–15	No	Easy	56 × 2	DU × U	Difficult	N/A
47–22	No	Easy	61 × 3	DU × U	Difficult	N/A
52–39	Yes	Difficult	72 × 2	DU × U	Difficult	N/A
43–27	Yes	Difficult	29 × 5	DU × U	Difficult	N/A
65–39	Yes	Difficult	45 × 4	DU × U	Difficult	N/A
54–18	Yes	Difficult	31 × 6	DU × U	Difficult	N/A

U, one-digit number; DU, two-digits number.

### Statistical analysis

**FIGURE 1:**
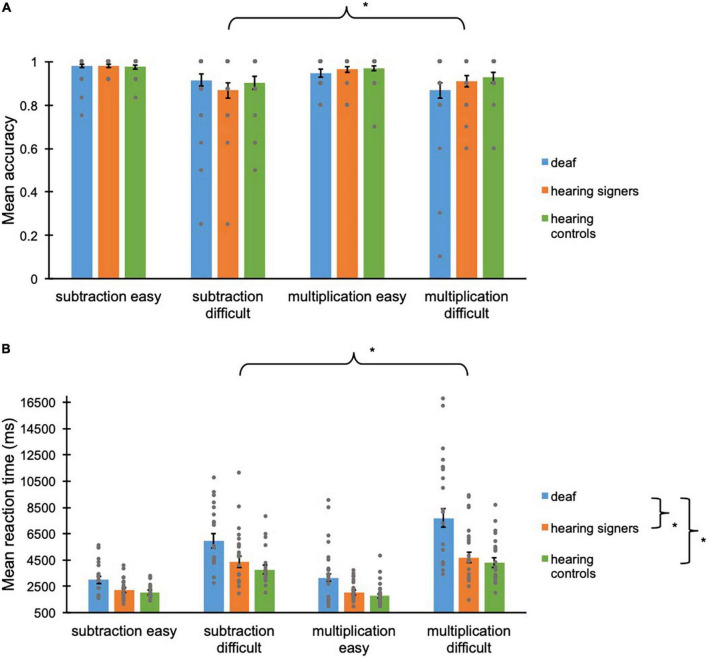
**(A)** Mean accuracy scores (proportions) and **(B)** mean reaction times (ms) for deaf (in blue), hearing signers (in orange), and hearing controls (in green) for the two different operations, and the two different levels of the arithmetic task. Error bars represent the standard error of the means. Asterisks represent significant difference. Grey points represent individual mean scores.

Statistical analyses were carried out using IBM SPSS statistics 26 software for Mac OS Monterey 12.0.1 (Armonk, NY, USA). Statistical significance was set at *p* < 0.05 for all computations. Data were checked for normality of distribution and presented as Mean ± Standard Error (SE). Accuracy scores and reaction times (in ms) were measured. A binary Generalised Linear Mixed Model (GLMM) was run on accuracy scores (correct or not correct). A GLMM was run on the reaction time data (only reactions times for correct answers were included), indicating gamma distribution. One random factor was included in all analyses because of its significant contribution to the variance (i.e., subjects). The fixed factors included *Group* (deaf, hearing signers, hearing controls), *Operation* (subtraction, multiplication) and *Level* (easy, difficult), as well as their interactions. Given that deaf adults often experience executive functioning difficulties due to language deprivation, we hypothesised that our deaf group might be more sensitive to interference. To investigate if deaf individuals are indeed more affected by the interference index of single-digit multiplication problems, we performed a GLMM indicating gamma distribution with *Group*, *Interference index* and its interaction as fixed factors, and reaction time as dependent variable. Interference indexes were taken from De Visscher and Noël (2014b), since the authors calculated the interference index for all the 36 single-digit multiplication problems. As this index is not prone to change, and always remains the same for one specific operation, we could use this value directly in our analysis (see Figure 1 of De Visscher and Noël, 2014b; Table 2 for the related interference index of the single-digit multiplications presented in this study). Sequential Bonferroni adjusted significance level was applied when appropriate. Only the first and last model of the GLMM analyses where all non-significant interactions were not considered anymore in the model are reported, to (1) obtain a model that is quite easy to interpret, and (2) to gain power for the remaining parameters to detect significance. Outlier data were removed from statistical analysis when 3 standard deviations out of the mean (i.e., one deaf participant was removed from the testing sample). For subtraction problems, 0.95% outlier data were removed for accuracy and for reaction times in the deaf group; 0.48% for accuracy and 2.38% for reaction times in the hearing signer group, and 1.90% of the reaction times for the hearing controls group. For multiplication, the proportion of outliers for accuracy was 1.20% for the deaf group, 0.24% for the hearing signers, and 0.48% for the hearing controls. A total of 2.38% was removed of the reaction times for the hearing signer as well as for the hearing control group, and 2.62% for the deaf group.

## Results

### Accuracy

A binary GLMM was run on the accuracy scores as described above. No main effect of *Operation* [*F*_(1, 2494)_ = 2.55; *p* = 0.11] or *Group* [*F*_(2, 2494)_ = 0.13; *p* = 0.88] was shown. Nevertheless, a significant main effect of *Level* [*F*(_1, 2494)_ = 57.8; *p* < 0.001] and a significant *Level x Operation* interaction was observed [*F*_(1, 2494)_ = 4.42; *p* = 0.036]. The *Group* × *Operation* [*F*_(2, 2494)_ = 2.36; *p* = 0.095], *Group* × *Level* [*F*_(2, 2494)_ = 0.27; *p* = 0.77], and *Group* × *Operation* × *Level* [*F*_(2, 2494)_ = 0.23; *p* = 0.80] interactions were not significant. The final GLMM was run with the only significant interaction included and led to the same conclusion: Significantly higher accuracy scores were observed for the easy operations (*m* = 0.97, *se* = 0.005) compared to the difficult operations (*m* = 0.90, *se* = 0.015, *p* < 0.001). A significant difference between subtraction problems and multiplication problems was found for the easy operations only, where the accuracy scores of multiplication problems were lower (*m* = 0.96, *se* = 0.009) than those for subtraction problems (*m* = 0.98, *se* = 0.005, *p* = 0.025) (see Figure 1A).

### Reaction times

Regarding the reaction times, the GLMM indicated a significant difference for *Operation* [*F*_(1, 2265)_ = 4.26; *p* = 0.039], *Group* [*F*_(2, 2265)_ = 10.1; *p* < 0.001], and *Level* [*F*_(1, 2265)_ = 938; *p* < 0.001]. No significant *Group* × *Level* interaction [*F*_(2, 2265)_ = 0.32; *p* = 0.72] and no *Group* × *Operation* × *Level* interaction [*F*_(2, 2265)_ = 0.51; *p* = 0.60] was seen. However, significant *Group* × *Operation* [*F*_(2, 2265)_ = 4.00; *p* = 0.018] and *Operation* × *Level* [*F*_(1, 2265)_ = 16.4; *p* < 0.001] interactions were observed. The final GLMM was run including the two significant interactions and led to the same conclusion: Subtraction problems (*m* = 3318, *se* = 162) were solved faster than multiplication problems (*m* = 3492, *se* = 170, *p* = 0.040). Deaf adults (*m* = 4551, *se* = 372) were slower than hearing signer adults (*m* = 3114, *se* = 254, *p* = 0.003), and hearing control adults (*m* = 2783, *se* = 227, *p* < 0.001). Hearing signers did not perform differently compared to hearing controls (*p* = 0.33). Responses to difficult operations were slower (*m* = 4987, *se* = 246) than responses to easy operations (*m* = 2323, *se* = 112, *p* < 0.001). The difference between deaf (*m* = 4917, *se* = 417 *for multiplication problems and m* = 4213, *se* = 355 *for subtraction problems*) and hearing signers (*m* = 3122, *se* = 263 *for multiplication problems*, *p* = 0.001 and *m* = 3106, *se* = 262 *for subtraction problems*, *p* = 0.024) as well as between deaf and hearing controls (*m* = 2775, *se* = 234 *for multiplication problems*, *p* < 0.001 and *m* = 2791, *se* = 236 *for subtraction problems*, *p* = 0.003) was bigger for the multiplication problems than for the subtraction problems. The difference between subtraction problems and multiplication problems was only found for the difficult operations (*m* = 5379, *se* = 280 *for multiplication problems and m* = 4623, *se* = 248 *for subtraction problems*, *p* < 0.001), and not for the easy ones (*m* = 2267, *se* = 117 *for multiplication problems and m* = 2381, *se* = 120 *for subtraction problems*, *p* = 0.13) (see Figure 1B). Similar results are found when including years of formal education as covariate (see Supplementary material). Moreover, no speed accuracy trade-off was observed in any groups and/or any operations.

**FIGURE 2:**
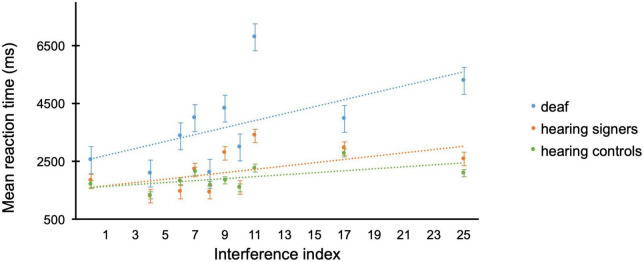
Mean reaction times (ms) per interference index for the easy multiplication operations in the different groups (deaf in blue, hearing signers in orange, hearing controls in green) of the arithmetic task. Error bars represent the standard error of the means.

When investigating the interference index using a GLMM, a main effect of *Group* [*F*_(2, 561)_ = 7.96; *p* < 0.001], and *Interference index* [*F*_(9, 561)_ = 23.0; *p* < 0.001] was observed together with a significant *Group* × *Interference index* interaction [*F*_(18, 561)_ = 2.10; *p* = 0.005]. Deaf (*m* = 3010, *se* = 309) performed slower than hearing signers (*m* = 1975, *se* = 202), *p* = 0.011, and hearing controls (*m* = 1727, *se* = 177), *p* = 0.001 (see Figure 2). *Post-hoc* analyses on the *Group* × *Interference index* interaction can be found as Supplementary Table 3. In general, a pattern indicating more significant group differences with augmenting interference index was observed.

## Discussion

Deafness has been indicated as a risk factor for mathematical difficulties, where the differences between signed and spoken language, less exposure to numerical language, and differences in domain-general processing are suggested to contribute mostly to this phenomenon (see Santos and Cordes, 2022 for a review). The challenges that deaf individuals experience with mathematical abilities have indeed been consistently demonstrated in the literature over the last decades (e.g., Wollman, 1965; Hine, 1970; Wood et al., 1986; Bull, 2008), and are thought to primarily lie in the acquisition of verbal number concepts such as counting, fractions, and, more importantly for our purposes, arithmetic skills (Titus, 1995; Leybaert and Van Cutsem, 2002; Kritzer, 2009; Pagliaro and Kritzer, 2013). While the underperformance of deaf individuals in arithmetic has been highlighted by different mathematical assessment tests (Bull et al., 2011 for math achievement test; Hyde et al., 2003 for arithmetic word problems; Kelly et al., 2003 for relational statements; Pagliaro and Ansell, 2012 for arithmetic story problems), the differential impact deafness and language deprivation may have on verbal vs. visuospatial arithmetic operations is less clear. To examine this possible dissociation, deaf signer, hearing signer and hearing control adults were asked to solve easy vs. difficult subtraction and multiplication operations.

Overall, our results demonstrated that performances were lower for the difficult operations as compared to the easy ones, and lower for multiplication problems as compared to subtraction problems in all three groups. This accuracy difference between subtraction problems and multiplication problems was, however, only present for the easy operations, while the reaction time difference between the same operations was only present for the difficult operations. But, most importantly for our purposes, and in contrast to Andin et al. (2019), we managed to highlight a difference between deaf signers and hearing adults at the behavioural level. Group differences were found for reaction times–but not for accuracy scores–(i.e., the deaf were slower than the two hearing groups), and these group differences were larger for multiplication problems than for subtraction problems. The discrepancy between our study and the one of Andin et al. (2019) probably comes from the fact that different groups of participants and different tasks were tested in these two studies. Indeed, in Andin et al. (2019), the deaf participants group only included native signers. Participants were moreover required to verify (and not calculate) the results of subtraction and multiplication problems. In a verification task, individuals can decide that the answer is false on the basis of plausibility judgements (e.g., Duverne and Lemaire, 2004, 2005; Hinault et al., 2015). Solution times are therefore not representative of the genuine time it takes to solve an arithmetic operation in an ecological situation. It is finally worth mentioning that only single-digit operations were included in this study. This level of arithmetic reasoning might have not been sufficient enough to highlight group differences in adults (Andin et al., 2022).

Recent years have seen a surge in empirical studies examining the role of language in accounting for cross-language disparities in children’s number understanding and arithmetic competence (Fuson and Kwon, 1992; Rasmussen et al., 2006; Wang et al., 2008; Krinzinger et al., 2011; Göbel et al., 2014). It has for example been suggested that the superior arithmetic performance of Chinese and other Asian students could be explained by the relative *linguistic transparency* of the Asian counting systems (Fuson and Kwon, 1992; Miller et al., 2005), which gives a clear and consistent representation of the base-ten system (contrarily to the base-five system of the sign languages used in Belgium). In line with this, when considering bilingual individuals, the language in which arithmetic was learned seems to have a remaining advantage on performance. Van Rinsveld et al. (2016), for example, found better performances on arithmetic problem solving in German than in French, since German is the first learned language at the Luxembourgish school system. While comparisons across different auditory languages have been made, the present study aimed to examine the impact of sign language use on arithmetic problem solving. Since the obtained results indicated no significant difference between hearing signer and hearing control adults, one could assume that it is rather the usage of sign language as L1, while having learned multiplication in spoken language, that influences multiplication performances and not the knowledge of sign language *per se*. Belgian deaf signers could possibly use a visuospatial route while solving multiplication operations. Hearing individuals would in contrast directly access the verbal route. The visuospatial detour that deaf signers experience could explain why solving multiplication operations requires them more time and resources (i.e., cognitive load). Hearing individuals may not prevent themselves from relying on the phonological aspects of the presented stimuli, while deaf signers may experience some issues in accessing the verbal associations of multiplication facts. This hypothesis is, however, speculative and should be further tested in the future.

As the control groups (hearing controls and hearing signers) have experienced typical language development with typical language access from birth, they differ from the deaf group in language modality and in hearing status but also in early language access. This delay in accessing language can therefore be the main factor subtending the arithmetic difficulties of our deaf sample. Signed languages are indeed complete, natural languages that consist of their own unique visual grammar and syntax (Stokoe et al., 1965). Consistently with the fact that typically developing children with higher phonological awareness are better in forming verbal representations of multiplicative relations between two numbers (De Smedt et al., 2010; Berteletti et al., 2014), deaf children born to deaf parents who are fluent signers, do not display the same difficulties with mathematics as those with language deprivation early in development (e.g., Kritzer, 2009; Mousley and Kurz, 2015; Hrastinski and Wilbur, 2016). This distinction highlights an important relationship between language access and acquiring numerical concepts, or the importance of mastering sign language phonology to perform well on multiplication in the deaf signers population (e.g., Berteletti et al., 2022). Supplementary Figure 1 representing individual data indicates that the early deaf signers seem to be more efficient than the later deaf signers of our sample. Late deaf signers probably experienced some early language deprivation and possibly limited access to spoken languages during the critical years for learning mathematics. Speculatively, if all opted to acquire sign language later in life (i.e., after the age of 3 years/o), it is probably because the quality of the auditory input or the difficulty in processing it was non-negligible (see Supplementary Figure 1). More systematically comparing native or late signers to early or late cochlear implanted deaf individuals would definitely help to understand whether the mathematical difficulties deaf often experience originate from auditory deprivation *per se* or from a delay in accessing and mastering verbal or visual languages.

While Andin et al. (2019) failed to find behavioural group differences in reaction time and accuracy on their arithmetic task, they nevertheless highlighted differences in the neural networks deaf signers and hearing non-signers engage to calculate (but see Andin et al., 2022; Berteletti et al., 2022). Whereas language related brain regions in the left cerebral hemisphere are usually recruited for arithmetic fact retrieval (Dehaene et al., 2003), stronger activation of the right horizontal intraparietal sulcus was found in deaf signers compared to hearing non-signers. This indicates that deaf signers may solve multiplication operations by relying on magnitude manipulation to a larger extent than their hearing peers (Andin et al., 2019). They could therefore be more sensitive to the numerical magnitude of the operations presented (see Supplementary Figures 2, 3). In line with this, we have to admit that our multiplication problems tend to be larger in overall magnitude than our subtraction problems. The greatest difficulty that we observe in deaf signers for the multiplication problems may therefore lie in their greater quantity processing rather than to the visuo-phonemes translation they require. If small multiplication problems (Siegler, 1988) are solved by direct memory retrieval, it is true that larger multiplication problems are more likely to be split up in easier problems and then involve visuospatial procedures to manipulate intermediate calculations and the magnitude of the final result (LeFevre et al., 1996; Thevenot et al., 2001, 2007; Núñez-Peña et al., 2011). As splitting up the operation in easier problems involves retrieving them as arithmetic facts, we are nevertheless convinced that multiplication problems (easy and difficult) require more language and memory processes than subtraction operations. Retrieving arithmetic facts and manipulating intermediate calculations could therefore be difficult for deaf individuals. This makes even more sense if we consider that: (1) language deprivation correlates with executive functioning difficulties (Hall et al., 2016; Botting et al., 2017; Jones et al., 2020; Ribner et al., 2022); and (2) spoken language is temporal and has been shown to lead to higher serial spans than signed information in serial recall tasks (Bavelier et al., 2008). As deaf signers who present language deprivation were shown to perform significantly poorer on executive functioning tasks than hearing individuals (Figueras et al., 2008; Hauser et al., 2008; Hintermair, 2013; Dye and Hauser, 2014; Hall et al., 2016; Botting et al., 2017; Jones et al., 2020), they could therefore heighten more sensitivity to interference. This assumption was supported by our interference index analysis. Moreover, as temporal order is maintained more efficiently in auditory-based representations than in visually-based representations (Paivio and Csapo, 1971; Watkins and Watkins, 1980; Watkins et al., 1992), speakers would rely more on temporal encoding, while signers would rely more on spatial encoding (Wilson, 2001). Deaf individuals could therefore experience more problems to solve multiplication operations, as they might be less efficient to learn a sequence of multiplication facts.

To conclude, our findings are in line with several previous studies suggesting that deaf individuals have no deficits in their numerical representation of magnitude information (i.e., similar accuracy scores), but might experience a less efficient processing (i.e., slower reaction times) of basic numerical information (Epstein et al., 1994; Iversen et al., 2004; Bull et al., 2005; Chinello et al., 2012; Rodriguez-Santos et al., 2014). This less efficient processing is, in our case, more pronounced for multiplication than for subtraction operations and could be explained by several mutually not exclusive reasons: (1) the fact that deaf individuals have delayed and therefore less automatic access than their hearing peers to the verbal phonological loop (Elliott et al., 2011); (2) the fact that deaf individuals might show higher sensitivity to the magnitude of the arithmetic operation presented; and (3) the fact that deaf individuals might show higher sensitivity to interference for multiplication operations (De Visscher and Noël, 2013, 2014a,b; De Visscher et al., 2018).

## Conclusion

This study investigated how deafness and its related variable language experience, including language deprivation, shapes verbal vs. visuospatial arithmetic performances. Although the accuracy scores between deaf signers, hearing signers and hearing controls did not differ, the deaf signers showed significantly slower reaction times compared to the two hearing groups. Importantly, this significant group difference was larger for multiplication operations than for subtraction operations. These findings support the idea that numerical tasks relying on verbal processes are more strongly impacted by deafness and its following language experience, compared to numerical tasks implying visuospatial processes (Buyle et al., 2022). Further studies are, however, needed to better understand the mechanisms underlying this dissociation. Performances of deaf and hearing children should for example be compared on many more “easy” operations. Varying much more the magnitude of the operands and the interference index of the multiplications presented will help to understand the impact of these two factors on the arithmetic development of deaf individuals. Asking deaf and hearing children to perform multiplication operations under verbal vs. visuospatial load may similarly help to understand whether deaf and hearing signers use different processes to solve these operations (verbal processes in hearing vs. visuospatial processes or magnitude manipulation in deaf signers).

## Data availability statement

The raw data supporting the conclusions of this article will be made available by the authors, upon request to the corresponding author.

## Ethics statement

The studies involving human participants were reviewed and approved by the “Comité d’Ethique Hospitalo-Facultaire Saint-Luc-UCLouvain” (2019/19AOU/357). The patients/participants provided their written informed consent to participate in this study.

## Author contributions

MB: conceptualisation, methodology, software, formal analysis, investigation, data curation, visualisation, writing – original draft and review, and editing. VC: conceptualisation, methodology, software, writing – review and editing, supervision, validation, project administration, and funding acquisition. Both authors contributed to the article and approved the submitted version.
