# Supplementary material for: Deafness and early language deprivation influence arithmetic performances
Source: Front Hum Neurosci. 2022 Nov 30;16:1000598. doi: 10.3389/fnhum.2022.1000598 (PMC9748281; doi:10.3389/fnhum.2022.1000598)
Supplement: Supplementary file 1 [file Data_Sheet_1.docx]

Supplementary Material

# Supplementary Data

Although indeed great variations exist in Belgium regarding education for deaf individuals and also great variation exists in our deaf sample, performing the analysis including education (i.e., formal school years after primary school) as a covariate did not indicate the significant contribution of education years on the obtained results: A GLMM indicating gamma distribution and considering years of education as covariate resulted in following significant differences: *Group* [*F(2, 2208) = 8.89; p < .001*], and *Level* [*F(1, 2208) = 909; p < .001*]. *Operation* [*F(1, 2208) = 3.53; p = .061*] was marginally significant. There was no significant contribution of *Education* [*F(1, 2208) = .635; p = .43*]. No significant *Group x Level* interaction [*F(2, 2208) = .39; p = .68*] and no *Group* x *Operation* x *Level* interaction [*F(2, 2208) = .57; p = .56*] was seen. However, a marginally significant *Group* x *Operation* [*F(2, 2208) = 2.84; p = .059*] and significant *Operation* x *Level* [*F(1, 2208) = 15.2; p < .001*] interactions were observed. The final GLMM was run including the two (marginally) significant interactions and led to a similar conclusion indicating also a significant *Group* x *Operation* [*F(2, 2212) = 3.21; p = .041*] interaction now: Deaf adults (*m = 4449, se = 377*) were slower than hearing signer adults (*m = 3067, se = 261, p = .006*), and hearing control adults (*m = 2774, se = 226, p < .001*). Hearing signers did not perform differently compared to hearing controls (*p = .40*). Responses to difficult operations were slower (*m = 4913, se = 246*) than responses to easy operations (*m = 2295, se = 112, p < .001*). The difference between deaf (*m = 4762, se = 418 for multiplication problems and m = 4156, se = 363 for subtraction problems*) and hearing signers (*m = 3084, se = 270 for multiplication problems, p = .002 and m = 3051, se = 268 for subtraction problems, p = .032*) as well as between deaf and hearing controls (*m = 2767, se = 233 for multiplication problems, p < .001 and m = 2782, se = 234 for subtraction problems, p = .004*) was bigger for the multiplication problems than for the subtraction problems. A difference between subtraction problems and multiplication problems was only found for the difficult operations (*m = 5283, se = 279 for multiplication problems and m = 4569, se = 248 for subtraction problems*, *p < .001*), and not for the easy ones (*m = 2237, se = 117 for multiplication problems and m = 2354, se = 120 for subtraction problems, p = .12*).

# Supplementary Figures and Tables

Supplementary Table 1. *Sign Language Knowledge* *in Hearing Signer Participants*

| Subject | Learned sign language at | Frequency of use |
| --- | --- | --- |
| 23 | 6 y/o | Daily |
| 24 | 20 y/o | Daily |
| 25 | 18 y/o | Daily |
| 26 | 28 y/o | N/A |
| 27 | As a kid | Weekly |
| 28 | 18 y/o | Daily |
| 29 | 18 y/o | Daily |
| 30 | N/A | Daily |
| 31 | 28 y/o | Weekly |
| 32 | 26 y/o | Daily |
| 33 | 18 y/o | Daily |
| 34 | 22 y/o | Weekly |
| 35 | Implicit 10 y/o, explicit 25 y/o | Weekly |
| 36 | 26 y/o | Weekly |
| 37 | 19 y/o | Weekly |
| 38 | 22 y/o | Weekly |
| 39 | 4-5 y/o | Weekly |
| 40 | 18 y/o | Weekly |
| 41 | 32 y/o | Weekly |
| 42 | 28 y/o | Weekly |
| 43 | As a baby | Daily |

Supplementary Table 2. *Language and Hearing Aid Characteristics of Deaf Participants*

| Subject | Deafness level | Hearing aids | Hearing aids use since | Sign language knowledge | French/Dutch level | Sign language use frequency | Mother tongue | Sign language learned at |
| --- | --- | --- | --- | --- | --- | --- | --- | --- |
| 1 | profound | yes | 2 y/o | yes | good | daily | French | 16 y/o |
| 2 | profound | yes | 6 y/o | yes | good | daily | Dutch | 7 y/o |
| 3 | severe | yes | 7 y/o | yes | excellent | daily | French | 5 y/o |
| 4 | profound | CI | 6 y/o | yes | Secondary school level | daily | LSFB | Mother tongue |
| 5 | profound | no | / | yes | fairly good | daily | VGT | Mother tongue |
| 6 | profound | CI | 2005 | yes | excellent | daily | French | 20 y/o |
| 7 | Profound and severe | CI | 16 y/o | yes | good | daily | French | 19 y/o |
| 8 | profound | used to have it, not anymore | 5 y/o right ear | yes | bad | daily | VGT | Native |
| 9 | profound | yes | 1 y/o but stopped wearing it at 16 y/o | yes | normal | always | LSFB | Native |
| 10 | profound | Yes, left ear | 5 y/o | yes | good | daily | VGT | Mother tongue |
| 11 | profound | CI, right ear | 2,5 y/o | yes | good | daily | VGT | Mother tongue |
| 12 | profound | Yes, but does not use it often; left ear | / | yes | moderate | always | VGT | Native |
| 13 | Profound right ear and severe left ear | yes | 8 months | yes | good | daily | French | 14 y/o |
| 14 | profound | CI | 2 y/o | yes | bon | daily | French | 2 y/o |
| 15 | profound | no | / | yes | does not speak | always | VGT | Native |
| 16 | profound | no | / | yes | moderate | always | VGT | Native |
| 17 | profound | yes, left ear | 3 y/o | yes | good | daily | VGT | Mother tongue |
| 18 | profound | no | / | yes | bad | daily | Dutch with gestures | 51 y/o |
| 19 | profound | yes, left ear | since primary school | yes | weak | daily | VGT | Native |
| 20 | Profound and severe | yes, right ear | 11 months | yes | bon | daily | LSFB | Native |
| 21 | profound | CI | left ear 2,5 y/o; 13 y/o right ear | yes | N/A | daily | French | 2 y/o |
| 22 | profound | CI right, but does not use it often | 2 y/o | yes | good | daily | LSFB | Mother tongue |

Supplementary Table 3. *Post-hoc Analyses Group x Interference index Interaction of GLMM*

| Interference Index | Group | P-value |
| --- | --- | --- |
| 0 | D vs HS | .47 |
| 0 | D vs HC | .47 |
| 0 | HS vs HC | .86 |
| 4 | D vs HS | .077 |
| 4 | D vs HC | .077 |
| 4 | HS vs HC | .97 |
| 6 | D vs HS | .008* |
| 6 | D vs HC | .008* |
| 6 | HS vs HC | .90 |
| 7 | D vs HS | .60 |
| 7 | D vs HC | .14 |
| 7 | HS vs HC | .60 |
| 8 | D vs HS | .33 |
| 8 | D vs HC | .39 |
| 8 | HS vs HC | .76 |
| 9 | D vs HS | .13 |
| 9 | D vs HC | .003* |
| 9 | HS vs HC | .067 |
| 10 | D vs HS | .27 |
| 10 | D vs HC | .27 |
| 10 | HS vs HC | .93 |
| 11 | D vs HS | .007* |
| 11 | D vs HC | .000* |
| 11 | HS vs HC | .051 |
| 17 | D vs HS | .20 |
| 17 | D vs HC | .071 |
| 17 | HS vs HC | .51 |
| 25 | D vs HS | .008* |
| 25 | D vs HC | .001* |
| 25 | HS vs HC | .33 |

*Note.* D = deaf, HS = hearing signers, HC = hearing controls, * = significant.

## Supplementary Figures

**Supplementary Figure 1.** Mean reaction times (ms) for early deaf signers (blue) and later deaf signers (orange) for the two different operations, and the two different levels of the arithmetic task. We had 14 early deaf signers in our sample (i.e., before the age of 3) versus 7 later signers. Grey points represent individual mean scores. Visually we can observe that early deaf signers seem to have faster reaction times compared to later deaf signers overall. The reds points on the graph indicate the individual data of the native deaf signers (born in a deaf family, n=7), and the green points indicate the individual data of early implanted (i.e., before the age of 3 y/o) deaf (n=4). Interestingly, we can see from these points that although their distribution lies rather spread among the individual data points, the majority of the red points (i.e., native deaf signers) nevertheless seem to indicate rather faster reaction times compared to the green (i.e., early implanted) data points. The difference between the red and the green data points tends to be even more pronounced for the difficult operations. In conclusion, the data seems to indeed indicate a tendency towards better arithmetic performances for early deaf signers.

**Supplementary Figure 2.** Mean reaction times (ms) for deaf signers (blue), hearing signers (orange), and hearing controls (green) for the “easy” multiplication operations, presented by their outcome. As illustrated in the graph, a typical size effect (i.e., reaction times are longer when problems are presented with larger operands, and thus larger answers) can be observed for all groups.

**Supplementary Figure 3.** Mean reaction times (ms) for deaf signers (blue), hearing signers (orange), and hearing controls (green) for the “easy” subtraction operations, presented by their outcome. The graph illustrates a typical size effect for all groups.
